# Construction of energy-conserving sucrose utilization pathways for improving poly-γ-glutamic acid production in *Bacillus amyloliquefaciens*

**DOI:** 10.1186/s12934-017-0712-y

**Published:** 2017-06-06

**Authors:** Jun Feng, Yanyan Gu, Yufen Quan, Weixia Gao, Yulei Dang, Mingfeng Cao, Xiaoyun Lu, Yi Wang, Cunjiang Song, Shufang Wang

**Affiliations:** 1 Key Laboratory of Molecular Microbiology and Technology for Ministry of Education, Nankai University, Tianjin, 300071 China; 2 Key Laboratory of Biomedical Information Engineering of Ministry of Education, School of Life Science and Technology, Xi’an Jiaotong University, Xi’an, 710049 Shaanxi China; 3 Department of Biosystems Engineering, Auburn University, Auburn, AL 36849 USA; 4 State Key Laboratory of Medicinal Chemical Biology, Nankai University, 94 Weijin Road, Tianjin, 300071 China; 5 Department of Chemical and Biological Engineering, Iowa State University, Ames, IA 50011 USA

**Keywords:** *Bacillus amyloliquefaciens*, Sucrose utilization pathway, Phosphoenolpyruvate (PEP)-dependent phosphotransferase system (PTS), Energy-conserving pathway, Sucrose permease, Sucrose phosphorylase, Poly-γ-glutamic acid (γ-PGA)

## Abstract

**Background:**

Sucrose is an naturally abundant and easily fermentable feedstock for various biochemical production processes. By now, several sucrose utilization pathways have been identified and characterized. Among them, the pathway consists of sucrose permease and sucrose phosphorylase is an energy-conserving sucrose utilization pathway because it consumes less ATP when comparing to other known pathways. *Bacillus amyloliquefaciens* NK-1 strain can use sucrose as the feedstock to produce poly-γ-glutamic acid (γ-PGA), a highly valuable biopolymer. The native sucrose utilization pathway in NK-1 strain consists of phosphoenolpyruvate-dependent phosphotransferase system and sucrose-6-P hydrolase and consumes more ATP than the energy-conserving sucrose utilization pathway.

**Results:**

In this study, the native sucrose utilization pathway in NK-1 was firstly deleted and generated the *B. amyloliquefaciens* 3Δ strain. Then four combination of heterologous energy-conserving sucrose utilization pathways were constructed and introduced into the 3Δ strain. Results demonstrated that the combination of *cscB* (encodes sucrose permease) from *Escherichia coli* and *sucP* (encodes sucrose phosphorylase) from *Bifidobacterium adolescentis* showed the highest sucrose metabolic efficiency. The corresponding mutant consumed 49.4% more sucrose and produced 38.5% more γ-PGA than the NK-1 strain under the same fermentation conditions.

**Conclusions:**

To our best knowledge, this is the first report concerning the enhancement of the target product production by introducing the heterologous energy-conserving sucrose utilization pathways. Such a strategy can be easily extended to other microorganism hosts for reinforced biochemical production using sucrose as substrate.

**Electronic supplementary material:**

The online version of this article (doi:10.1186/s12934-017-0712-y) contains supplementary material, which is available to authorized users.

## Background

Sucrose is composed of a glucose unit linked to a fructose unit by the glycosidic bond. It is the most abundant disaccharide available in the nature [1]. Sucrose is mostly obtained from sugarcane juice or sugar beet [2]; Recently, attempts for utilization of sucrose as a cheap and easily degradable feedstock for biochemical production through microbial fermentation has attracted a lot of interests [3, 4].

**Fig. 1:**
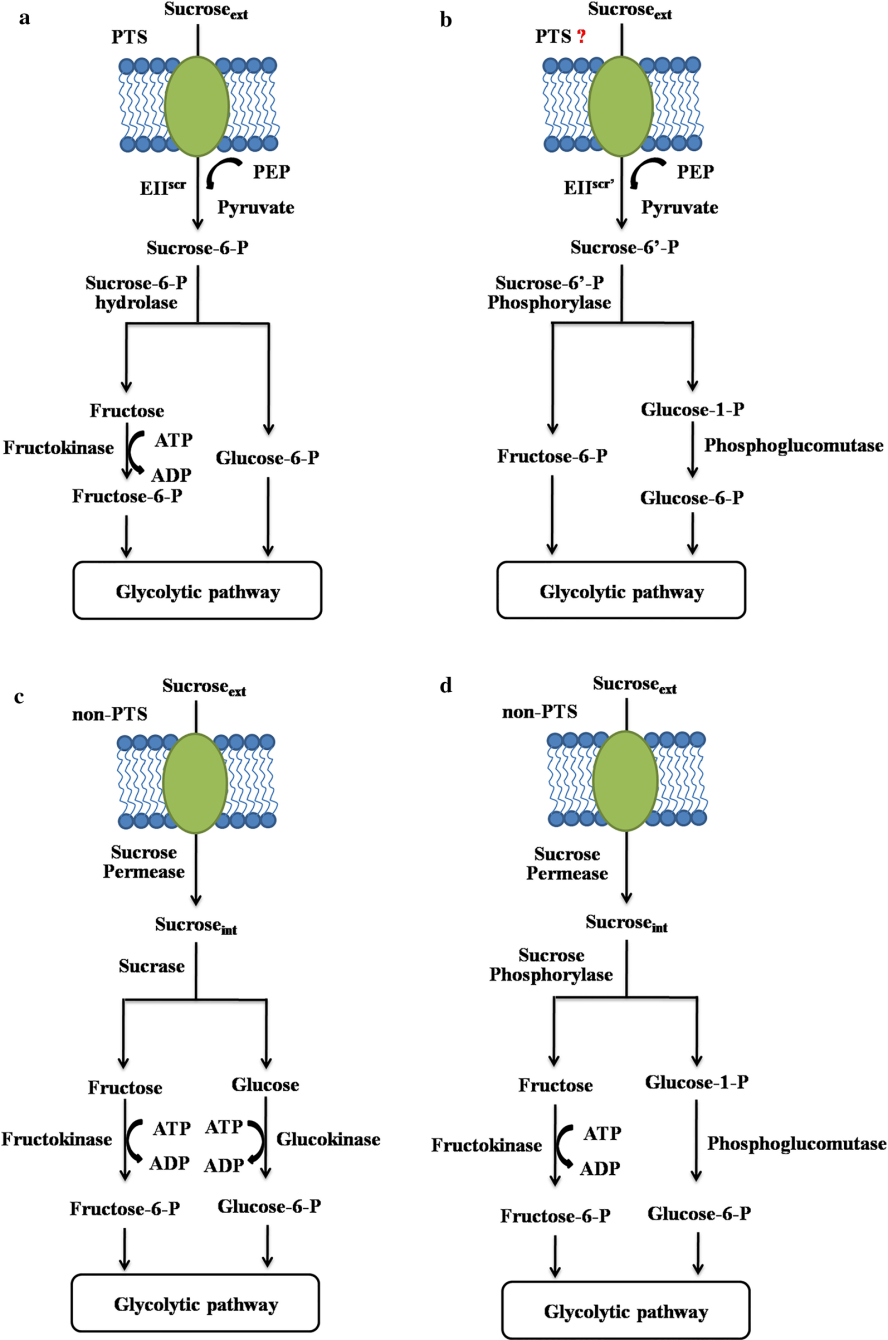
Possible pathways for sucrose utilization in nature. **a** Sucrose utilization pathway consists of PTS and sucrose-6-P (β-d-Fru-(2 → 1)-α-d-Glc 6-P) hydrolase; **b** sucrose utilization pathway consists of a putative PTS system and sucrose-6′-P (β-d-Fru 6-P-(2 → 1)-α-d-Glc) phosphorylase; **c** non-PTS (non-phosphotransferase system) sucrose utilization pathway consists of sucrose permease and sucrase; **d** non-PTS sucrose utilization pathway consists of sucrose permease and sucrose phosphorylase

There are several possible metabolic pathways for sucrose utilization (Fig. 1). They can be divided into two groups based on two different sugar transport mechanisms: phosphoenolpyruvate (PEP)-dependent phosphotransferase system (PTS) [5] and non-PTS [6]. In PTS, sucrose is transported across the cytoplasmic membrane via a sucrose-specific PEP-dependent phosphotransferase, while in non-PTS, sucrose is taken up by the sucrose permease [7].

Figure 1a shows a PTS associated sucrose utilization pathway. The extracellular sucrose is transported into the cell by the sucrose-specific PEP-dependent phosphotransferase and converted to sucrose-6-P (β-d-Fru-(2 → 1)-α-d-Glc 6-P). The obtained sucrose-6-P is then hydrolyzed by sucrose-6-P hydrolase, generating fructose and glucose-6-P. The fructose is afterwards catalyzed by ATP-dependent fructokinase to generate fructose-6-P. The obtained fructose-6-P and glucose-6-P are then entering the glycolytic pathway. Figure 1b shows a hypothetic sucrose utilization pathway that may exist in nature. Verhaeghe et al. [8] reported a new sucrose phosphorylase, named sucrose-6′-P phosphorylase, which can catalyze sucrose-6′-P (β-d-Fru 6-P-(2 → 1)-α-d-Glc) into fructose-6-P and glucose-1-P. They speculated that the sucrose-6′-P phosphorylase represented a new pathway for sucrose utilization. They hypothesized that, along with the sucrose-6′-P phosphorylase, a new sucrose-specific PTS can attach a phosphate to the fructose moiety other than the glucose moiety. Then the generated sucrose-6′-P along with the sucrose-6′-P phosphorylase constituted a new sucrose utilization pathway. As shown in Fig. 1c, d, is the non-PTS sucrose utilization pathways and the extracellular sucrose is transported into the cell by sucrose permease. The intracellular sucrose is then hydrolyzed by sucrase (Fig. 1c) or sucrose phosphorylase (Fig. 1d). Sucrase hydrolyzes sucrose into fructose and glucose; while sucrose phosphorylase catalyzes the reversible phosphorolysis of sucrose in the presence of inorganic phosphate, to yield glucose-1-P and fructose [9]. The glucose is catalyzed by ATP-dependent glucokinase to generate glucose-6-P and the fructose is catalyzed by fructokinase to generate fructose-6-P. The glucose-1-P can be converted to glucose-6-P by the phosphoglucomutase (Fig. 1d). Finally, both products (glucose-6-P and fructose-6-P), as intermediates, enter the glycolytic pathway. When comparing the ATP consumption for the conversion of one molecule sucrose into fructose-6-P and glucose-6-P, we can easily notice that through the pathway depicted in either Fig. 1b or d, only one molecule of ATP is needed, while for the pathways illustrated in Fig. 1a or c, two molecules of ATP are needed. For Fig. 1a, b, one molecular PEP is converted into pyruvate when one molecular sucrose is transformed into sucrose-6-P; thus it can be counted as one molecular ATP consumption.

Because the pathway as shown in Fig. 1b has not yet been reported, the pathway depicted in Fig. 1d is the currently available most energy-conserving sucrose utilization pathway to our best knowledge. We thus hypothesized that, when sucrose is used as the substrate, the overexpression of the energy-conserving sucrose utilization pathway in the host strain can enhance the sugar consumption and also the relevant bioproduct production.

**Fig. 2:**
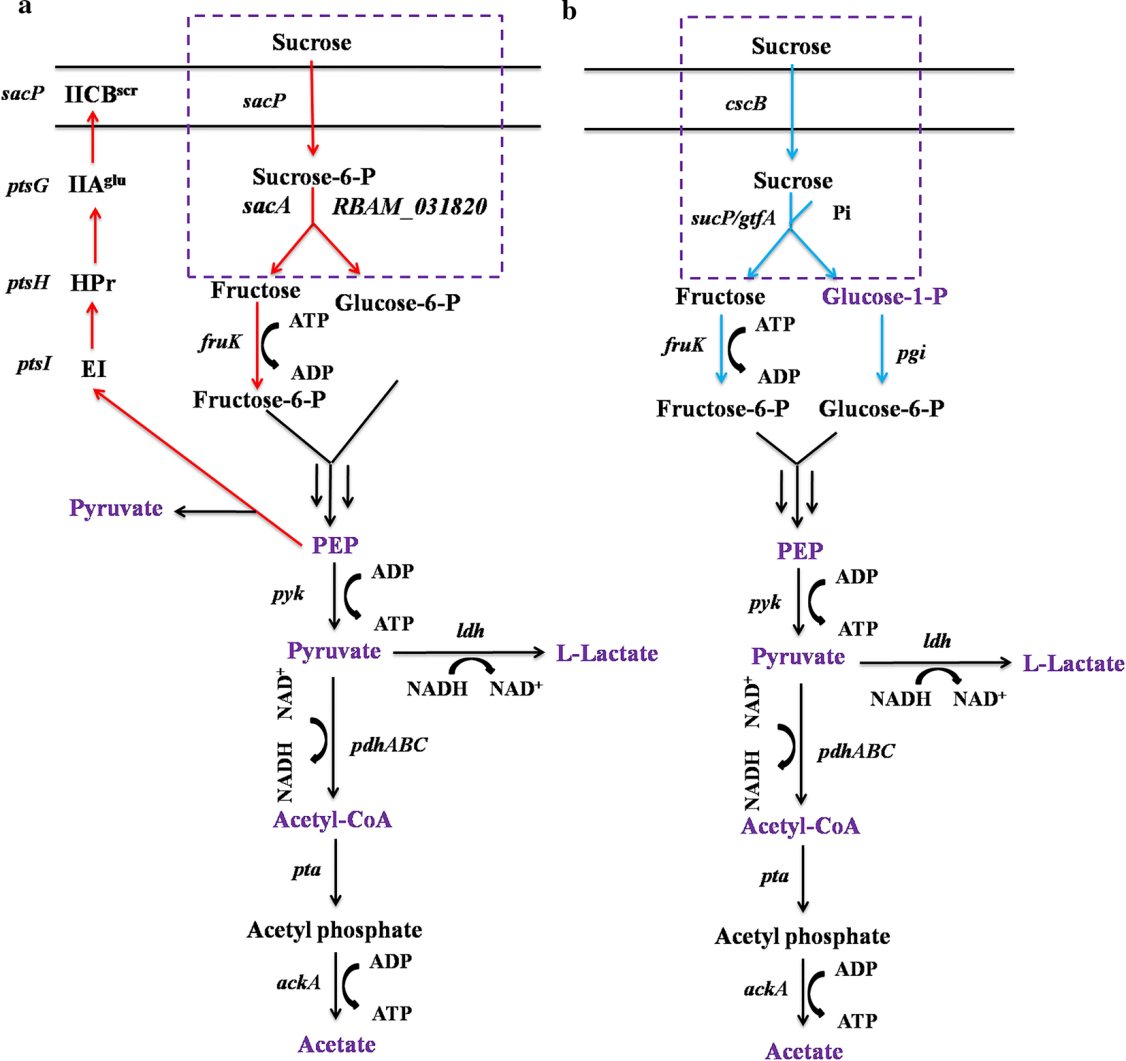
Schematic for the replacement of the sucrose utilization pathway in *Bacillus amyloliquefacien*s NK-1 strain. **a** The native sucrose utilization pathway in NK-1; **b** the to-be-introduced heterologous energy-conserving sucrose utilization pathway. *sacP*, PTS sucrose-specific enzyme IIBC component; *ptsG*, PTS glucose-specific EIIA component; *ptsH*, phosphocarrier protein HPr; *ptsI*, PTS enzyme I; *sacA*, sucrase-6-phosphate hydrolase; *RBAM_031820*, sucrase-6-phosphate hydrolase; *pyk*, pyruvate kinase; *cscB*, sucrose permease; *sucP*, sucrose phosphorylase; *gtfA*, sucrose phosphorylase; *fruK*, fructose kinase; *pgcA*, phosphoglucomutase; *ldh*, lactic dehydrogenase; *pta*, phosphotransacetylase; *pfhABC*, pyruvate dehydrogenase; *ackA*, acetate kinase. The* purple words* indicated the metabolites measured in this work

Poly-γ-glutamic acid (γ-PGA) is an important, naturally occurring polyamide consisting of d/l-glutamate monomers [10]. It exhibits many favorable features such as biodegradable, water soluble, edible and non-toxic to humans and the environment. Therefore, it has been widely used in fields of foods, medicines, cosmetics and agriculture [11]. *Bacillus amyloliquefaciens* NK-1 strain is a glutamate-independent poly-γ-glutamic acid (γ-PGA) producing strain, and it can use sucrose as the carbon source [12–14]. The whole genome of this strain has been sequenced [15]. The genome indicated that the strain consumes sucrose through the sucrose-specific PTS along with the sucrose-6-P hydrolase (Fig. 1a). As shown in Fig. 2a, the sucrose-specific PTS is composed of non-sugar specific enzyme I (EI) and HPr (encoded by genes *ptsI* and *ptsH*, respectively), and sucrose-specific enzyme II^scr^ complex containing the soluble IIA^Glc^ enzyme (encoded by *ptsG*) and the integral membrane permease IICB^Scr^ (encoded by *sacP*) [5]. A phosphate group is transferred from PEP to EI and HPr, then to soluble protein IIA^Glc^ and IICB^Scr^ that can recognize and transport the sucrose molecules [16, 17]. One molecule PEP is required for transportation and phosphorylation of one molecule sucrose [5], and the generated sucrose-6-P is then catalyzed by sucrose-6-P hydrolase encoded by *sacA* and *RBAM_031820*. In addition, in the *B. amyloliquefaciens* genome, the *sacB* gene encodes a second sucrose-hydrolyzing enzyme called levansucrase, which can catalyze sucrose into glucose and levan [18]. The generated glucose can also be used for cell growth.

In this study, our objective was to enhance the sucrose metabolism and γ-PGA production in *B. amyloliquefaciens* by introducing the heterologous energy-conserving sucrose utilization pathway. We first attempted to block the native sucrose utilization pathway in NK-1 strain (including the sucrose-specific PTS associate pathway genes and *sacB*). Then, four combinations of the energy-conserving sucrose utilization pathways consisting of the sucrose permease gene (*cscB* from *Escherichia coli* or *cscB* from *Bifidobacterium lactis*) and the sucrose phosphorylase gene (*sucP* from *Bifidobacterium adolescentis* or *gtfA* from *Streptococcus mutans*) were introduced into the sucrose-utilization-deficient strain (Fig. 2b). To our best knowledge, this is the first report concerning the enhancement of sucrose metabolism in the host strain by overexpressing the heterologous energy-conserving sucrose utilization pathway in a *Bacillus* strain.

## Methods

### Microorganisms, plasmids and cultivation conditions

All the strains and plasmids used in this work are listed in Table 1. All the *B. amyloliquefaciens* and *E. coli* strains were grown at 37°C in Luria–Bertani (LB) medium for routine strain construction and maintenance. For *B. amyloliquefaciens* γ-PGA production, fermentation was carried out in the γ-PGA fermentation medium, which contains: 50 g/L sucrose, 6 g/L (NH_4_)_2_SO_4_, 0.6 g/L MgSO_4_, 6 g/L KH_2_PO_4_, 14 g/L K_2_HPO_4_ and trace elements with 1 mM of FeSO_4_, CaCl_2_, MnSO_4_ and ZnCl_2_ [12]. One milliliter seed culture was transferred into 100 mL γ-PGA fermentation medium in the 500 mL shaking flasks to start the fermentation. The fermentation was performed at 37 °C, with an agitation rate of 180 rpm for 48 h. When necessary, antibiotics were used at the following concentrations: 100 μg/mL ampicillin, 5 μg/mL chloramphenicol and 20 μg/mL tetracycline. The concentration of 5-fluorouracil used for mutant selection was 100 μg/mL. The *B. amyloliquefaciens* NK-1 strain is a derivative of LL3 strain, which is deposited in the China Center for Type Culture Collection (CCTCC) with accession number CCTCC M 208109 [15].

**Table 1:** Strains and plasmids used in this study

Strains and plasmids	Relevant genotype and characteristics	Source
Strains		
*B. amyloliquefaciens* NK-1	LL3 derivative, ΔpMC1, Δ*upp*	[18]
*B. amyloliquefaciens* NK-1-Δ*stpa*	NK-1 derivative, Δ*sacTPA*	This work
*B. amyloliquefaciens* Δ1	NK-1 derivative, Δ*sacA*	This work
*B. amyloliquefaciens* Δ2	NK-1 derivative, Δ*sacA*, Δ*sacBlevB*	This work
*B. amyloliquefaciens* Δ3	NK-1 derivative, Δ*sacA*, Δ*sacBlevB*, Δ*RBAM_031820*	This work
*B. amyloliquefaciens* Δ1-CES	Δ1 derivative with expression plasmid pWH1520-CES	This work
*B. amyloliquefaciens* Δ1-CEG	Δ1 derivative with expression plasmid pWH1520-CEG	This work
*B. amyloliquefaciens* Δ1-CBS	Δ1 derivative with expression plasmid pWH1520-CBS	This work
*B. amyloliquefaciens* Δ1-CBG	Δ1 derivative with expression plasmid pWH1520-CBG	This work
*B. amyloliquefaciens* Δ2-CES	Δ2 derivative with expression plasmid pWH1520-CES	This work
*B. amyloliquefaciens* Δ2-CEG	Δ2 derivative with expression plasmid pWH1520-CEG	This work
*B. amyloliquefaciens* Δ2-CBS	Δ2 derivative with expression plasmid pWH1520-CBS	This work
*B. amyloliquefaciens* Δ2-CBG	Δ2 derivative with expression plasmid pWH1520-CBG	This work
*B. amyloliquefaciens* Δ3-CES	Δ3 derivative with expression plasmid pWH1520-CES	This work
*B. amyloliquefaciens* Δ3-CEG	Δ3 derivative with expression plasmid pWH1520-CEG	This work
*B. amyloliquefaciens* Δ3-CBS	Δ3 derivative with expression plasmid pWH1520-CBS	This work
*B. amyloliquefaciens* Δ3-CBG	Δ3 derivative with expression plasmid pWH1520-CBG	This work
*E. coli* DH5α	F^−^, φ80d*lac*ZΔM1, Δ(*lacZYA*-*argF*)U169, *deoR*, *recA*1, *endA*1, *hsdR*17(r_k_^−^, m_k_^+^), *phoA*, *supE*44, λ^−^ *thi*-1, *gyrA*96, *relA*1	Lab stock
*E. coli* GM2163	F^−^, *ara*-*14 leuB6 thi*-*1 fhuA31 lacY1 tsx*-*78 galK2* *galT22 supE44 hisG4 rpsL 136 (Str* ^*r*^ *) xyl*-*5 mtl*-*1* *dam13::*Tn9 (Cam^r^) *dcm*-*6 mcrB1 hsdR2 mcrA*	Lab stock
Plasmids		
p-KSU	pKSV7-derivation with *upp* gene	[20]
pKSV7-ΔsacA	p-KSU-derivation with deletion fragment of *sacA*	This work
pKSV7-Δsac	p-KSU-derivation with deletion fragment of *sac* operon	[33]
pKSV7-ΔRBAM_031820	p-KSU-derivation with deletion fragment of *RBAM_031820*	This work
pKSV7-ΔsacTPA		
pWH1520	Tc^r^; xylose inducible expression vector for *Bacillus*	MoBiTec
pWH1520-CES	pWH1520 derivative with genes *cscB* (*E. coli*) and *sucP*	This work
pWH1520-CEG	pWH1520 derivative with genes *cscB* (*E. coli*) and *gtfA*	This work
pWH1520-CBS	pWH1520 derivative with genes *cscB* (*B. lactis*) and *sucP*	This work
pWH1520-CBG	pWH1520 derivative with genes *cscB* (*B. lactisi*) and *gtfA*	This work

### DNA manipulation, plasmid construction and strain construction

The plasmids for gene deletion (pKSV7-ΔsacA, pKSV7-ΔRBAM_031820 and pKSV7-ΔsacTPA) were constructed as previously reported protocols [14]. The gene deletion in this study was carried out following a previously reported marker-less gene deletion method [19, 20]. All the primers used in this study were listed in Additional file 1: Table S1.

**Fig. 3:**
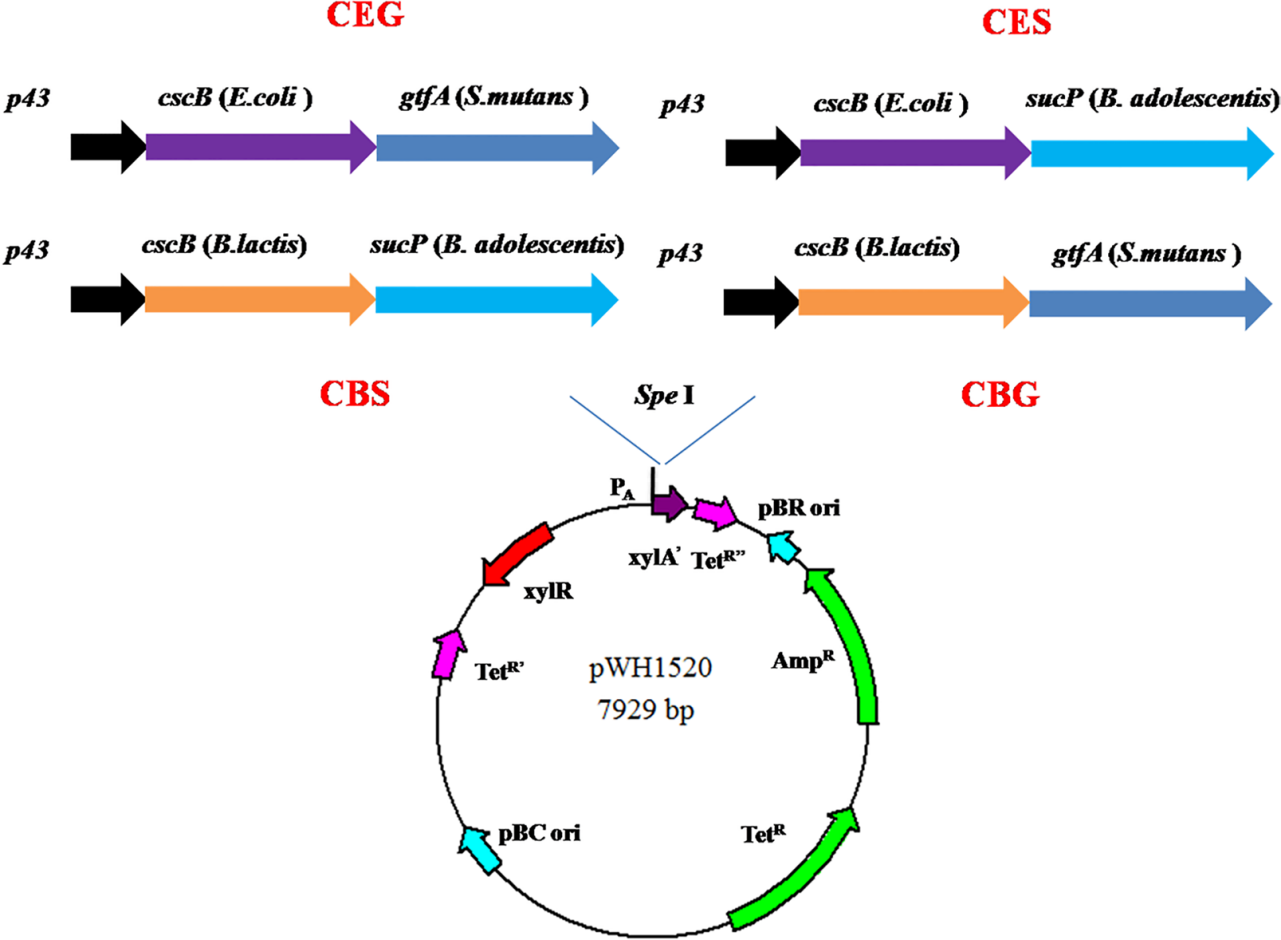
Construction of the energy-conserving sucrose utilization pathways. The four combinations of pathways: CEG [*cscB* (*E. coli*) + *gtfA* (*S. mutans*)], CES [*cscB* (*E. coli*) + *sucP* (*B. adolescentis*)], CBS [*cscB* (*B. lactis*) + *sucP* (*B. adolescentis*)] and CBG [*cscB* (*B. lactis*) + *gtfA* (*S. mutans*)] were put under P_43_ promoter and expressed by pWH1520 plasmid

The two sucrose permease genes and sucrose phosphorylase genes were codon optimized to match the *Bacillus* spp codon usage and synthesized by Genscript (Nanjing, China). All the optimized gene sequences were listed in Additional file 2: Table S2. The P_43_ promoter and the synthesized genes were amplified by PCR using PrimeSTAR HS DNA polymerase (Takara Bio, Japan). The three DNA fragments (P_43_ promoter, sucrose permease gene and sucrose phosphorylase gene) were joined together by overlapping-PCR. The generated fragments were digested and ligated into the *Spe*I site of pWH1520 vector, generating expression plasmids pWH1520-CES, pWH1520-CEG, pWH1520-CBS and pWH1520-CBG, respectively (Fig. 3).

The *sacTPA* cluster in NK-1 strain was deleted and the obtained strain was designated as *B. amyloliquefaciens* NK-1-Δ*stpa* The *sacA*, *sacB* and *RBAM_031820* gene (encoding sucrose-6-phosphate hydrolase) were deleted in turn in the NK-1 strain and the obtained strains were designated as *B. amyloliquefaciens* 1Δ, *B. amyloliquefaciens* 2Δ and *B. amyloliquefaciens* 3Δ, respectively.

As a naming rule in this study, after each of the four expression plasmids pWH1520-CES, pWH1520-CEG, pWH1520-CBS and pWH1520-CBG was transformed into each of the NK-1, 1Δ, 2Δ, 3Δ strains, the finally generated strains were designated as N-CES, N-CEG, N-CBS and N-CBG correspondingly (N represents the corresponding host strain; for example, strains generated from *B. amyloliquefaciens* 1Δ were named as *B. amyloliquefaciens* 1Δ-CES, 1Δ-CEG, 1Δ-CBS, 1Δ-CBG, respectively).

### Metabolites assays

The metabolites (glucose-1-phosphate, PEP, pyruvate, acetate, lactate, acetyl-CoA and ATP) from NK-1, 3Δ, 3Δ-CES, 3Δ-CEG, 3Δ-CBS and 3Δ-CBG strains were measured in this work. Each strain was cultivated in the γ-PGA fermentation medium for 36 h. The cultures were collected, and the cells were pelleted by centrifugation (4 °C 8000 rpm for 20 min). The supernants were used for lactate and acetate measurement (see below). The cell pellets were washed three times with PBS buffer (pH 7.0) and then adjusted to the same optical density (OD) for all the samples by PBS buffer. The cells were then broken by a sonicator (600 W for 30 min with cycles of 3 s sonication followed by 3 s pause). The broken cells were centrifugated at 12,000 rpm for 3 min and the supernants were used for the measurement of glucose-1-phosphate, PEP, pyruvate, acetyl-CoA and ATP.

The concentrations of acetate were measured by high-performance liquid chromatography (HPLC) with the BDS 5 u column (Alltech, USA) and the UVIS 200 detector (Alltech, USA). (NH_4_)H_2_PO_4_ (0.04 mol/L, pH 2.6) was used as the mobile phase with a flow rate of 1 mL/min at 35 °C. The concentration of lactate was measured using a lactate analyzer (SBA-40D, Biology Institute of Shandong Academy of Sciences, China). The concentrations of glucose-1-phosphate, PEP, pyruvate, acetyl-CoA and ATP were all measured using commercial assay kits following the manufacturer’s instructions. The concentration of glucose-1-phosphate was measured using the Glucose-1-Phosphate (G1P) Colorimetric Assay Kit (Sigma-Aldrich, USA). The concentration of PEP was measured using the PEP Colorimetric/Fluorometric Assay Kit (Sigma Aldrich, USA). The concentration of pyruvate was measured using the Pyruvic acid (PA) Colorimetric Assay Kit (Comin, China). The concentration of acetyl-CoA was measured using the Acetyl-CoA Colorimetric Assay Kit (Comin, China). The intracellular ATP was measured using the ATP Assay Kit (Beyotime Biotechnology, China).

### γ-PGA fermentation and analytical procedures

γ-PGA was purified using a previously described method [21]. Concentrations of glucose, fructose and sucrose, were measured using an HPLC system. One milliliter of culture was centrifuged at 8000 rpm for 20 min. The supernatant was filtered with the 0.45 µm filter prior to analysis with a prevail carbohydrate ES 5 u (4.6 mm × 250 mm) column (Alltech, USA) and a refractive index (RI) detector (Schambeck SFD GmbH, Germany). 75% acetonitrile was used as the mobile phase with a flow rate of 1 mL/min at 35 °C.

## Results and discussion

### Construction of the sucrose utilization deficient strain

Carbon metabolic pathways are essential for the synthesis of precursors, providing free energy (ATP), and redox-cofactor balancing. Engineering of carbon metabolism is often very useful for the optimization of the productivity, and yield of the desirable products. There are three known sucrose utilization pathways been reported previously (Fig. 1). Most of the bacteria, including *B. amyloliquefaciens* NK-1 strain, degrade sucrose through the PTS pathway as showed in Fig. 1a. In this pathway, two molecules of ATP will be consumed for the hydrolysis of one molecule sucrose into fructose-6-P and glucose-6-P. However, there are also microorganisms degrading sucrose through a non-PTS pathway and using sucrose phosphorylase for the further phosphorylation as depicted in Fig. 1d, in which only one molecule of ATP is needed for the conversion of one molecule sucrose into fructose-6-P and glucose-6-P [22]. It is apprent that such a pathway saves more energy for sucrose consumption when compared with the other two pathways (Fig. 1a, c). The replacement of the native PTS-based sucrose utilization pathway with the energy-conserving sucrose utilization pathway may enhance the sucrose metabolism and thus the bioproduct production in the host strain when sucrose is used as the substrate. Therefore, in this study, we aimed to replace the native sucrose utilization pathway in *B. amyloliquefacies* NK-1 strain with the energy-conserving sucrose utilization pathway and further evaluate its effect on the cellular metabolism and the γ-PGA production.

First, we attempted to construct a sucrose utilization deficient strain by eliminating the native sucrose utilization pathway in NK-1 stain. In *B. amyloliquefaciens* NK-1, the native sucrose-specific PTS pathway consists of the *sacTPA* cluster and a hypothetical sucrose-6-P hydrolase gene (*RBAM_03820*). The *sacTPA* cluster is responsible for the transportation of the extracellular sucrose, and the deletion of the cluster will block sucrose transport and thus its further degradation. In this case, *RBAM_03820* will not need to be deleted because sucrose-6-P would be absent in the *sacTPA* negative strain. Therefore, the *sacTPA* cluster was first deleted in this study and the obtained strain was designated as NK-1-Δ*stpa*. However, interestingly, in the following experiment, we noticed that the strain was not transformable with all the plasmids we tried, including pKSV7, pHT01 and pWH1520. Therefore, the NK-1-Δ*spta* strain cannot be used in the following experiment for further metabolic engineering work.

SacP is a membrane protein and the deletion of *sacP* is the most likely reason that affects the plasmid transformation. Therefore, we determined to delete only *sacA* and *RBAM_03820* genes. In this way, our goal to eliminate the sucrose utilization in the host strain can still be achieved, because the lack of sucrose-6-P hydrolase (due to the deletion of *sacA* and *RBAM_03820*) will also lead to the deficiency of sucrose utilization. *sacA* was deleted and the generated strain was designated as 1Δ. We confirmed that the 1Δ strain with only *sacA* deleted can be transformed with plasmids. This verified that SacP really affected plasmid transformation in this strain, but the reason was still unknown. We then further deleted *sacB* and *RBAM_03820* based on the 1Δ strain. The obtained strains were designated as 2Δ and 3Δ, respectively.

**Fig. 4:**
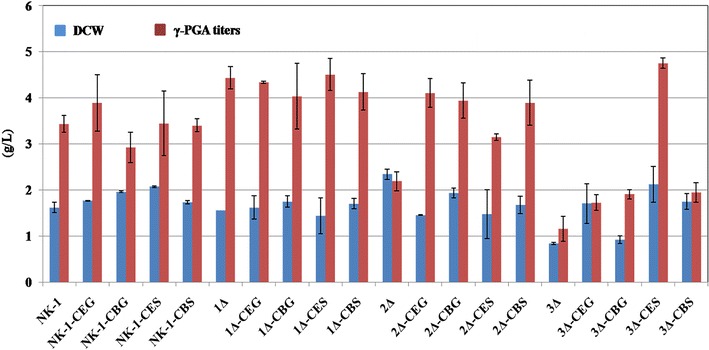
γ-PGA fermentation results of various strains. All the strains were cultured at γ-PGA fermentation medium for 48 h prior to the measurement of the dry cell weight (DCW) and γ-PGA prodcution. Values represent mean ± SD of triplicates

Fermentation was carried out using γ-PGA fermentation medium to characterize the mutants. Interestingly, as shown in Fig. 4, the 3Δ strain can still grow in the sucrose based medium. In the fermentation medium, beside the sucrose as the primary carbon source, small amounts of glucose (0.56 g/L) and fructose (0.39 g/L) were also detected; this might be the reason that the 3Δ strain can still survive in the sucrose-containing fermentation medium. However, its growth was severely impaired and the DCW was only about half of the NK-1 strain. Nevertheless, we believe that the 3Δ strain would be a good platform in which the heterologous energy-conserving sucrose pathways can be introduced and evaluated for enhanced sucrose metabolism and end product production. More detailed discussion about the phenotype of these mutants along with the mutants developed in the following steps are presented in the later sections.

### Construction of mutants with energy-conserving sucrose metabolic pathways

The energy-conserving non-PTS pathway consists two enzymes: sucrose permease and sucrose phosphorylase. Sucrose permease is a membrane-associate enzyme that is responsible for transporting the extracellular sucrose into the cell (Fig. 1d). It has been reported in both gram-negative and gram-positive bacteria [22]. It is well known that the membrane structure between gram-negtive microorganisms and gram-positve microorganisms are significantly different. To ensure the sucrose permease can function in the gram-positive *B. amyloliquefaciens*, we selected two sucrose permease genes for evaluation: a well characterized sucrose permease from the gram-negtive *E. coli* W strain (*cscB* (*E. coli*), WP_001197025.1; Fig. 3) [23] and a sucrose permease from the gram-positive *Bifidobacterium lactis* strain (*cscB* (*B. lactis*), CAD26969.1; Fig. 3) [24]. There have been many sucrose phosphorylases identified and characterized [25]. Aerts et al. [26] compared the transglucosylation potential of six sucrose phosphorylases towards different classes of acceptors and found that the sucrose phosphorylase from *Bifidobacterium adolescentis* [27, 28] and the one from *Streptococcus mutans* [29, 30] showed high activities for sucrose hydrolysis. Therefore we selected these two enzymes in this work. The sucrose permeases from *E. coli* W and from *Bifidobacterium lactis* and the sucrose phosphorylases from *Bifidobacterium adolescentis* (*sucP*, WP_011742626.1) and from *Streptococcus mutans* (*gtfA*, WP_002262875.1) were combined into four constructs representing the energy-conserving sucrose utilization pathways (Fig. 3). Each construct was put under the P_43_ promoter and expressed on the pWH1520 plasmid. Plasmids carrying these four constructs were designated as pWH1520-CEG, pWH1520-CES, pWH1520-EBS and pWH1520-CBG, respectively. Each plasmid was transformed into NK-1, 1Δ, 2Δ and 3Δ strains.

### Verification the intracellular function of the energy-conserving sucrose utilization pathways

**Fig. 5:**
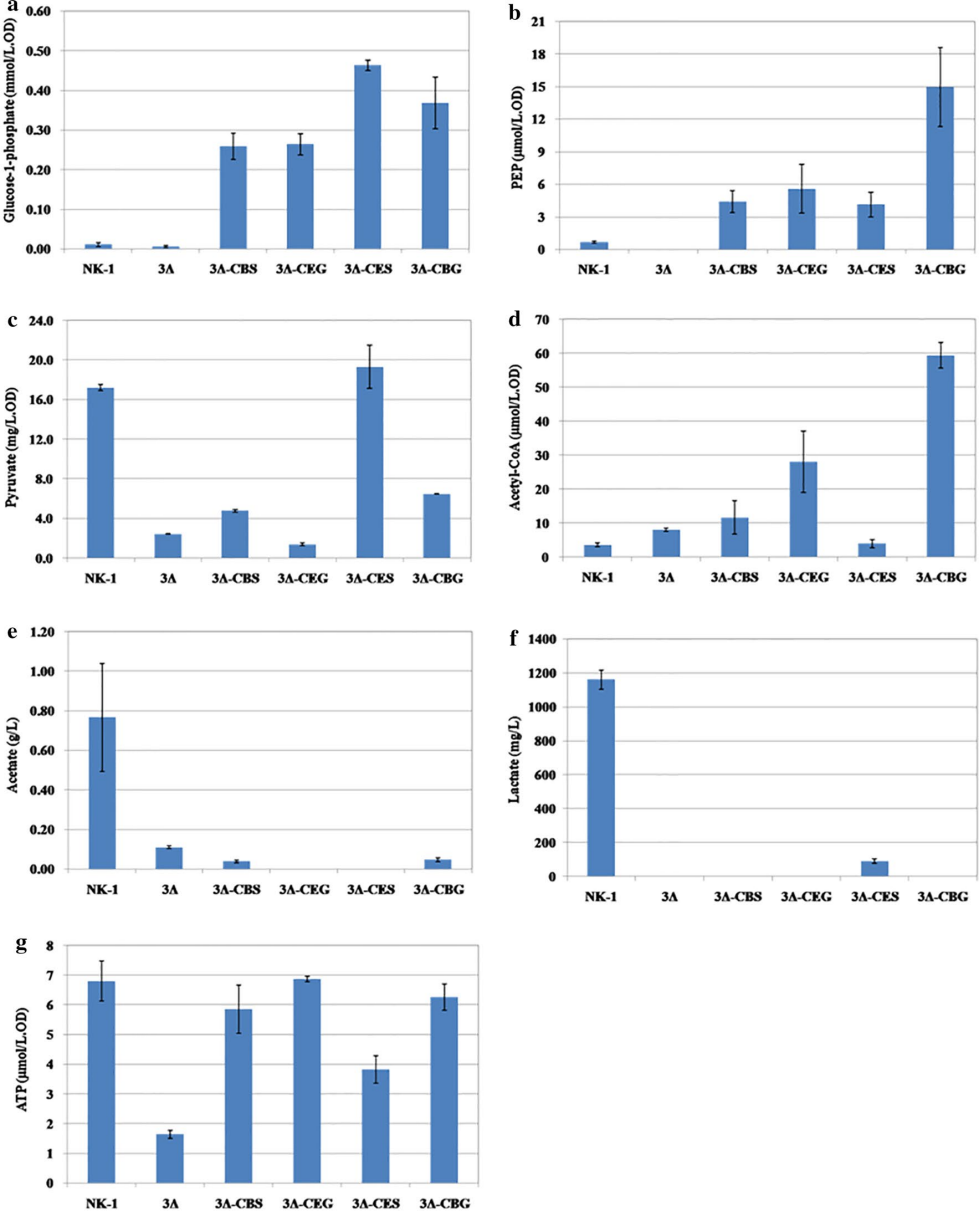
Measurement of metabolites associated with sucrose utilization in NK-1, 3Δ, 3Δ-CEG, 3Δ-CBG, 3Δ-CES and 3Δ-CBS strains. **a** Glucose-1-phosphate; **b** PEP; **c** Pyruvate; **d** Acetyl-CoA; **e** Acetate; **f** Lactate; **g** ATP

To verify the function of the heterologous pathways, the levels of metabolites associated with sucrose utilization were measured and compared between the wild-type NK-1 strain and the mutant strains (Figs. 2, 5). Glucose-1-P is a specific metabolite, which can only be synthesized in the energy-conserving pathway rather than in the native sucrose utilization pathway (Fig. 2). As seen in Fig. 5a, the intracellular glucose-1-P concentrations in the four mutant strains (3Δ-CEG, 3Δ-CES, 3Δ-CBS and 3Δ-CBG) were significantly higher than that in the NK-1 strain, indicating that the introduced energy-conserving pathways functioned well in *B. amyloliquefaciens*. The strain 3Δ-CES demonstrated the highest glucose-1-P concentration among the four mutant strains, which indicated that this strain has the highest intracellular sucrose hydrolytic activity. The negligible amount of glucose-1-P presented in the NK-1 strain might result from the reverse reaction between glucose-6-P and glucose-1-P by the phosphoglucomutase. PEP and pyruvate are another two important intermediate metabolites in the sucrose utilization pathways. In the native sucrose utilization pathway (Fig. 2a), part of the synthesized PEP will be used for the transportation of sucrose, during which pyruvate can also be generate. Thus, the intracellular PEP concentration in the NK-1 strain will be lower than that in the mutant strains containing heterologous sucrose utilization pathways, while the intracellular pyruvate concentration in NK-1 will be higher. As expected, all of the PEP concentrations were higher in the 3Δ-CEG, 3Δ-CES, 3Δ-CBS and 3Δ-CBG strains than that in the NK-1 strain (Fig. 5b). Except for the 3Δ-CES strain, all of the intracellular pyruvate concentrations in the other three mutant strains were lower than that in the NK-1 strain (Fig. 5c). Overall, the differences of the levels of these three metabolites (glucose-1-P, PEP and pyruvate) between the mutants and the wild type indicated that the introduced heterogenous sucrose utilization pathway functioned in the *B. amyloliquefaciens* strains.

Acetyl-CoA is an important intermediate in the central metabolic pathway; we also determined levels of acetyl-CoA in these various strains (Fig. 5d). Except for 3Δ-CES, the intracellular acetyl-CoA concentration in the other three mutant strains are higher than that in the NK-1 strain. Pyruvate and acetyl-CoA are very important central metabolites for cell metabolism and the balancing between them is very important for the normal cellular metabolism. Thus their intracellular amounts need to be strictly regulated. The 3Δ-CES strain has higher levels of glucose-1-P and PEP but comparable levels of pyruvate and acetyl-CoA than NK-1 strain. It seems that the 3Δ-CES strain can quickly adjust to the heterologous sucrose utilization pathway and the produced glucose-1-P, PEP and pyruvate can be quickly used to balance its cellular metabolism. This was further confirmed by the exceptional sucrose consumption and γ-PGA production of this strain as will be discussed in a later section.

The levels of two important byproducts (acetate and lactate) in the central metabolism were also measured. We found that the introduced energy-conserving sucrose utilization pathway led to significantly lower production of acetate and lactate. Only slight amounts of acetate and lactate were detected in their supernatants (Fig. 5e, f); both were reduced by >90% in the mutants when compared to the NK-1 strain. The reduction or elimination of byproducts (such as acetate and lactate particularly in this case) production are generally very effective strategies in metabolic engineering for desirable end products production; because this often leads more carbon flux for the synthesis of the target product [31, 32]. However, in this study, the reason why the introduction of energy-conserving sucrose utilization pathway resulted in the reduction of acetate and lactate production is unknown. Since we introduced the energy-conserving pathway for sucrose utilization, we were curious to see whether such manipulation could lead to the accumulation of intracellular ATP. As shown in Fig. 5g, interestingly, all the intracellular amounts of ATP were comparable among the four mutant strains and the NK-1 strain. It seems that the microorganism has robust regulatory mechanism to banlance the intracellular ATP at a dynamically stable level.

For comparison purpose, we also measured the relevant metabolite levels in the 3Δ strain. Since the 3Δ strain could not grow well under there conditions (Fig. 4), all the levels of these metabolites were lower than other strains (Fig. 5); especially the intracellular ATP concentration in the 3Δ strain was much lower than all the other strains (including the wild type NK-1 strain), indicating that the metabolic activity in the 3Δ strain was much more deficient than the other strains.

### Enhanced γ-PGA production in the mutants containing the energy-conserving sucrose utilization pathways

To verify the effects of energy-conserving sucrose utilization pathways on the sucrose metabolism and γ-PGA production, we characterized all the mutants comparing to the wild type through fermentation using the γ-PGA fermentation medium. We divided these strains into four groups: NK-1 group (NK-1, NK-1-CEG, NK-1-CBG, NK-1-CES and NK-1-CBS), 1Δ group (1Δ, 1Δ-CEG, 1Δ-CBG, 1Δ-CES and 1Δ-CBS), 2Δ group (2Δ, 2Δ-CEG, 2Δ-CBG, 2Δ-CES and 2Δ-CBS), and 3Δ group (3Δ, 3Δ-CEG, 3Δ-CBG, 3Δ-CES and 3Δ-CBS). As shown in Fig. 4, in the NK-1 group, the cell biomass production (DCW) in all the mutants were slightly higher than that in NK-1 strain, with NK-1-CES had a the highest DCW of 2.07 g/L vs. 1.62 g/L in the NK-1 strain. The γ-PGA production in mutant strains was comparable to that in the NK-1 strain, except that a slightly higher γ-PGA production (3.89 g/L) was observed in the NK-1-CEG strain than in the NK-1 strain (3.43 g/L). This might be because in these strains the native sucrose utilization pathway was complete and they primarily use the native pathway for sucrose consumption; while the heterologous sucrose utilization pathway had only marginal effects. In the 1Δ group, the cell biomass production were all comparable to each other, and also in the same range as that of NK-1 strain. The γ-PGA production in all the strains was higher than that of the NK-1 strain (17.5–31.5% higher than NK-1 strain depending on different strains). From our previous experience [33], we knew that the levan synthesized by levansucrase is one of the main byproducts in γ-PGA production. With the current γ-PGA purification protocol, the produced levan cannot be separated from the desirable γ-PGA. The γ-PGA purity increased from 83.2 to 91.5% after s*acB* was deleted [33]. with the deletion of *sacA* in this work, the 1Δ group strains mostly used *sacB* and *RBAM_031820* for sucrose metabolism and more levan was produced. Therefore, the increase in the γ-PGA production is mostly due to the impurity from the enhanced levan production. When *sacB* was further deleted in the 2Δ group, the 2Δ strain demonstrated increased cell growth, and all the other mutants had comparable cell growth as the NK-1 strain (with 2Δ-CEG and 2Δ-CES had slightly lower DCW than NK-1, and 2Δ-CBG and 2Δ-CBS had slightly higher DCW than NK-1). However, the higher cell growth of the 2Δ strain did not lead to corresponding higher γ-PGA production; actually its final γ-PGA level was lower than NK-1. 2Δ-CEG, 2Δ-CBG and 2Δ-CBS had 19.5, 14.9 and 13.4% higher γ-PGA production than NK-1, while the γ-PGA production in 2Δ-CES was slightly lower than NK-1. There is no levan production in the 2Δ group strains (as the impurity in γ-PGA), and thus the corresponding mutants in the 2Δ group showed lower γ-PGA production than that in the 1Δ group.

**Fig. 6:**
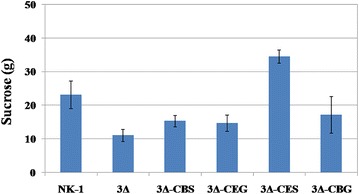
Sucrose consumption in NK-1, 3Δ, 3Δ-CEG, 3Δ-CBG, 3Δ-CES and 3Δ-CBS strains. All the strains were cultured in the γ-PGA fermentation medium for 48 h prior to measure their sucrose consumption. The results indicated the sucrose consumption amounts in 1 L medium. Values represent mean ± SD of triplicates

In the 3Δ group, all the genes related to the endogenous sucrose utilization pathway were deleted. However, the 3Δ strain could still survive in the sucrose medium, but its cell growth was significantly impaired. Besides the 3Δ strain, the sucrose consumption in 3Δ-CEG, 3Δ-CBG and 3Δ-CBS was much lower (25.2–36.5% lower depending on different strain) than that in the NK-1 strain, although a little bit higher than that in the 3Δ strain. This indicated that the introduced three heterologous sucrose utilization pathways were all functional in the corresponding strain, but their efficiency was not as good as the native PTS pathway. That might also be the reason why the γ-PGA production in these three strains was also lower (only about half of NK-1) than the control NK-1 strain (Fig. 5). The 3Δ-CBG strain showed a little bit higher DCW than the 3Δ strain but this DCW was only about 50% of the NK-1 strain; while 3Δ-CEG and 3Δ-CBS showed comparable or slightly higher DCW than the NK-1 strain. Remarkably, the 3Δ-CES strain showed the highest sucrose consumption, which was about 49.4% higher than that of the NK-1 strain (Fig. 6). The high sucrose consumption led to high DCW, which was 31.2% higher than the NK-1 strain (Fig. 4). The γ-PGA production in the 3Δ-CES strain was the highest among all the 20 strains and reached 4.75 g/L, which is 38.5% higher than that of the NK-1 strain. Interestingly, the strains containing the same heterologous sucrose utilization pathway (from NK-1, 1Δ, 2Δ and 3Δ groups) showed very different results. Especially in the NK-1 group and 2Δ group, the strains with CES didn’t show the best performance for γ-PGA production. The possible reason might be as follows: in these two groups of strains, they have the native sucrose utilization pathway. These strains can also use their native pathway for sucrose consumption. The γ-PGA production results are not the reflection of the introduced energy-conversing sucrose utilization pathway, but the reflection of the combination functions of the native and heterologous sucrose utilization pathways. It seems that the CES combination doesn’t exhibit its benefits with the existence of the native sucrose utilization pathway; in another words, in this case, the strains might still primarily use their native sucrose utilization pathway rather than the heterologous CES pathway. However, the 3Δ strain is a sucrose utilization deficient strain, and thus the γ-PGA production results are the reflection of only the heterologous energy-conversing sucrose utilization pathways. Thus, the results of the γ-PGA production from this group directly reflect the different performances of the various combinations of energy-conversing sucrose utilization pathways in the host strain. The results demonstrated that CES (sucrose permease from *E. coli* and sucrose phosphorylase from *Bifidobacterium adolescentis*) was the optimal among these four energy-conserving sucrose utilization pathways in terms of best sucrose metabolic efficiency for γ-PGA production in *B. amyloliquefaciens*. The introduction of energy-conserving sucrose utilization pathway not only increased sucrose metabolic efficiency but also boosted the production of the target product. This strategy can also be applied in other sucrose utilizing microorganisms in the future.

## Conclusion

In this study, we set out to enhance the sucrose metabolism in *B. amyloliquefaciens* for γ-PGA production by replacing the endogenous sucrose utilization pathway with the energy-conserving sucrose utilization pathways. We first deleted the PTS and sucrose-6-P hydrolase based sucrose utilization pathway in *B. amyloliquefaciens*, and then four heterologous energy-conserving pathways were constructed and introduced. Fermentation results demonstrated that the combination of *cscB* from *E. coli* and *sucP* from *Bifidobacterium adolescentis* showed the highest sucrose metabolic efficiency, which led to 49.4% higher sucrose consumption and 38.5% higher γ-PGA production in the generated mutant than the control strain. This is the first report concerning the enhancement of sucrose metabolism in the host strain by overexpressing the heterologous energy-conserving sucrose utilization pathway in a *Bacillus* strain. This strategy can be easily extended to other microorganisms for reinforced production of desirable bioproducts using sucrose as feedstock.

## Additional files

**Additional file 1: Table S1.** Primers used in this work.

**Additional file 2: Table S2.** Genes sequences used in this article.

## Abbreviations

γ-PGA: poly-γ-glutamic acid
PEP: phosphoenolpyruvate
PTS: phosphotransferase system
sucrose-6-P: sucrose-6-phosphate
Glucose-6-P: glucose-6-phosphate
Glucose-1-P: glucose-1-phosphate
Fructose-6-P: fructose-6-phosphate
ATP: adenosine triphosphate
ADP: adenosine diphosphate
SacB: levansucrase
