# Supplementary material for: Construction of energy-conserving sucrose utilization pathways for improving poly-γ-glutamic acid production in Bacillus amyloliquefaciens
Source: Microb Cell Fact. 2017 Jun 6;16:98. doi: 10.1186/s12934-017-0712-y (PMC5461702; doi:10.1186/s12934-017-0712-y)
Supplement: Supplementary file 1 — Additional file 1: Table S1. Primers used in this work. [file 12934_2017_712_MOESM1_ESM.pdf]

Table S1 Primers used in this study

Primers	Sequence (5' to 3')
sapt-SF	CCCCGGATCCTGAACAGAAGGTGGATGAAGACAGT
sapt-SR	CTACAAAAGTGTCCATTCTTGATAATCCATTATGTATCCC
sapt-XF	ATGGATTATCAAGAATGGACACTTTTGTAGAAAATCGTCCT
sapt-XR	CCCCGTCGACTCTATGCGGCAAAAGAATTTATCAC
sapt-SS	GTATTCGGCGATGAAGCATACAACC
sapt-XX	GATTGATACGCCTTACACACACGGA
sacA-SF	CCCCGGATCCGCGGGACTGGCTGTATTTTTCCTGACG
sacA -SR	CTACAAAAGTGTCCAGCCGTTCGGATCATTTAAAAGCCCG
sacA -XF	AATGATCCGAACGGCTGGACACTTTTGTAGAAAATCGT
sacA -XR	CCCCGTCGACTCTATGCGGCAAAAGAATTTATCAC
sacA -SS	TCGCTGATTTGGGCAGAAACTATATCC
sacA -XX	GATTGATACGCCTTACACACACGGA
S6P-SF	CCCCGGATCCTCAGCTTTTTTTCCAAATGTCTTTTAAC
S6P-SR	ATCGCGGAGACTGGTTGACATTGCGTGATGATCATAAACTTT
S6P-XF	CATCACGCAATGTCAACCAGTCTCCGCGATGCTTCCACAC
S6P-XR	CCCCGTCGACATGGATAGAATTCAGCAGGCGGAAGA
S6P-SS	TGCAGCATAATCCCGCATCATATAA
S6P-XX	CGCGTGCAGCATTTACAAAAAATC
CBS-SF	CCCCACTAGTTCCTTGTAGAGCTCAGCATTATTGAGTG
CBS-SR	TTGACATCTCCTTCTTTACATGTCTTCGCTATCTGCTGATA
CBS-XF	AGCGAAGACATGTAAAGAAGGAGATGTCAAAAATCAATGAAA
CBS-XR	CCCCACTAGTTTAGTGGTGGTGGTGGTGGTGTGCTACGACAGGCGGGT TGGCAATC
CBG-SF	CCCCACTAGTTCCTTGTAGAGCTCAGCATTATTGAGTG
CBG-SR	TATGTATCTCCTTTCTTTACATGTCTTCGCTATCTGCTGAT
CBG-XF	TAGCGAAGACATGTAAAGAAAGGAGATACATACAATGCCTAT
CBG-XR	CCCCACTAGTTTAGTGGTGGTGGTGGTGGTGTTCAAAGGAAATTGTCT

	GGTCGTTT
CES-SF	CCCCACTAGTTCCTTGTAGAGCTCAGCATTATTGAGTG
CES-SR	TTTGACATCTCCTTCTTTAGATAGCGCTAGGTACCGGCGTT
CES-XF	ACCTAGCGCTATCTAAAGAAGGAGATGTCAAAAATCAATGA
CES-XR	CCCCACTAGTTTAGTGGTGGTGGTGGTGGTGTGCTACGACAGGCGGGT TGGCAATC
CEG-SF	CCCCACTAGTTCCTTGTAGAGCTCAGCATTATTGAGTG
CEG-SR	ATGTATCTCCTTTCTTTAGATAGCGCTAGGTACCGGCGTTT
CEG-XF	CCTAGCGCTATCTAAAGAAAGGAGATACATACAATGCCTATTAT
CEG-XR	CCCCACTAGTTTAGTGGTGGTGGTGGTGGTGTTCAAAGGAAATTGTCT GGTCGTTT
