# Supplementary material for: Construction of energy-conserving sucrose utilization pathways for improving poly-γ-glutamic acid production in Bacillus amyloliquefaciens
Source: Microb Cell Fact. 2017 Jun 6;16:98. doi: 10.1186/s12934-017-0712-y (PMC5461702; doi:10.1186/s12934-017-0712-y)
Supplement: Supplementary file 2 — Additional file 2: Table S2. Genes sequences used in this article. [file 12934_2017_712_MOESM2_ESM.pdf]

Genes Sequences used in this article:

P_{43} promoter

TCCTTGTAGAGCTCAGCATTATTGAGTGGATGATTATATTCCTTTTGATAGGT

GGTATGTTTTCGCTTGAACTTTTAAATACAGCCATTGAACATACGGTTGATTT

AATAACTGACAAACATCACCCTCTTGCTAAAGCGGCCAAGGACGCTGCCG

CCGGGGCTGTTTGCGTTTTTGCCGTGATTTCGTGTATCATTGGTTTACTTATT

TTTTTGCCAAAGCTGTAATGGCTGAAAATTCTTACATTTATTTTACATTTTTA

GAAATGGGCGTGAAAAAAAGCGCGCGATTATGTAAAATATAAAGTGATAGC

GGTACCATTATAGGTAAGAGAGGAATGTACACATGAACAGACAAGAATTAA

TAACAGAAGCTT

***cscB* (*Escherichia coli* W)**

ATGGCACTGAACATCCCTTTTCGTAACGCCTACTATCGCTTTGCATCCTCCTA

TTCCTTTCTTTTTTTTATTTCCTGGTCACTGTGGTGGTCACTGTATGCCATTT

GGCTTAAAGGCCATCTGGGACTTACGGGTACGGAACTGGGAACACTTTACT

CAGTAAACCAATTTACGAGCATCTTGTTTATGATGTTTTACGGTATCGTGCA

GGATAAATTAGGCTTGAAAAAACCGCTTATCTGGTGCATGAGCTTTATTCTG

GTGCTTACAGGCCCGTTTATGATTTATGTCTACGAACCTCTGCTTCAATCTAA

TTTTTCCGTGGGTTTAATCTTGGGCGCGCTGTTTTTCGGTTTAGGCTATTTGG

CTGGATGTGGTTTGTTGGACTCTTTTACAGAAAAAATGGCCAGAAACTTTC

ATTTTGAATATGGAACGGCTCGCGCCTGGGGCTCATTTGGATACGCAATTGG

AGCATTTTTCGCTGGTATTTTCTTTTCAATCAGCCCGCATATCAACTTTTGGC

TGGTTTCACTTTTTGGCGCAGTGTTTATGATGATCAACATGAGATTTAAAGA

TAAAGACCATCAATGCGTCGCAGCGGATGCCGGCGGAGTTAAAAAAGAAG

ATTTTATCGCAGTGTTTAAAGACCGCAACTTTTGGGTTTTTGTGATCTTTATC

GTCGGAACATGGAGCTTTTACAACATCTTTGATCAACAGCTGTTTCCTGTAT

TTTACTCAGGCCTTTTTGAAAGCCATGACGTTGGCACGAGATTATATGGATA

CTTGAACTCTTTTCAGGTTGTGTTAGAAGCCTTGTGTATGGCAATTATCCCG

TTTTTCGTCAATCGTGTAGGCCCTAAAAACGCACTGCTTATCGGAGTCGTAA

TTATGGCGCTGCGGATTCTTTCTTGCGCTTTATTTGTTAATCCGTGGATTATC

TCCTTGGTGAAATTATTGCATGCGATCGAAGTCCCTCTTTGTGTTATCTCTGT

GTTTAAATACTCCGTCGCTAACTTTGATAAACGTTTGTCAAGCACAATTTTT

CTGATCGGTTTTCAAATCGCGTCTTCCCTGGGCATTGTTCTGCTTTCTACAC

CGACGGGAATTCTTTTTGACCATGCCGGTTATCAGACAGTTTTCTTTGCAAT

TTCCGGAATCGTTTGCCTTATGTTGTTGTTTGGCATTTTCTTTCTGAGCAAA

AAACGCGAACAGATTGTCATGGAAACGCCGGTACCTAGCGCTATCTAA

***cscB* (*Bifidobacterium lactis*)**

ATGGCGACGACAACGAAAGTTTGGCGGAATCCTTCTTATCTTCAATCCTCC

ACAGGCATCTTTTTATTTTTTTGCTCTTGGGGCATTTGGTGGTCATTTTTCCA

ACGTTGGTTGAATTCCATGGGACTGAACGGTGCCAAAGTTGGCACAATTTA

TTCTATCAATTCCCTTGCAACGCTTATTTTAATGTTTGGCTACGGATTAATCC

AAGATAACTTGGGCCTGAAAAGACGCCTTGTCTTAGTAATTTCAGCGATCG

CAGCGTTGGTGGGACCGTTTGTTCAGTTTGTGTATGCTCCTCTGATGAGAA

CAAATATGATGGCTGCCGCACTTGTGGGCTCTGTTGTGTTATCCGCTGGATT
TATGGCCGGTTGCTCTCTTATTGAACCGGTCACGGAACGTTATTCCCGTCGG
TTTAATTTAGAATACGGACAATCACGGGCGTGGGGCAGCTTTGGATATGCCA
TCGTCGCATTAGTAGCGGGCTTTGTCTTTAACATCAACCCGATGATCAACTT
TTGGCTGGGAAGCGCTTTTGGTGTCGGCATGCTTATTGTATATTTAACATGGT
ACCCGGCGGAACAGCGCGAAGCTCTTAAAGAAGCGGCTGATCCTAATGCC
GCACCGACAAACCCTACGATTAAAGACATGTTGGGCGTACTGAAAATGCCG
ACGCTTTGGGTTTTGATCGTGTTTATGCTGCTTACAAACACGTTTTACACAG
TTTTTGATCAACAGATGTTTCCGACGTATTACGCCAGCTTGTTTCCTAATGA
AGCAACAGGCAACGCGGTATACGGAACGCTTAATTCAGTTCAGGTGTTTTG
TGAAAGCGCCATGATGGGAGTCGTACCGATTATCATGCGTAAAGTCGGAGT
ACGGAACGCATTATTGCTGGGTTCTACAGTCATGTTTTTGAGAATTGGTCTG
TGCGGCATCTTTCATGATCCTGTTTCAATTAGCATCGTGAAAATGTTTCATGC
CATTGAAGTGCCGTTGTTTTGTCTGCCTGCATTTCGCTACTTTACACTTCATT
TTAACCCGAAACTTTCTGCGACGTTATACATGGTCGGCTTTCAAATTGCTTC
ACAAATCGGACAGGTTGTGTTTAGCACACCTCTTGGAATGTTACATGATAG
AATGGGCGACCGCACAACGTTTTTGACAATTTCAGCAATCGTCCTGGCGGC
TACGGTATACGGCTTTTTCGTTATCAAAAGAGATGACGAACAGGTTGATGG
AGACCCGTTTATCCGCGATTCTAAAAAATTGCCTTCCCTGGCTACAGACGA
AGCCATTTTATCAGCAGATAGCGAAGACATGTAA

gtfA

ATGCCTATTATCAACAAAACAATGCTTATCACATACGCTGACTCCTTGGGAA

AAAATCTGAAAGAACTGAACGAAAACATCGAAAATTATTTTGGCGATGCGG

TTGGCGGAGTGCATCTGCTTCCGTTTTTCCCTTCTACAGGAGATCGCGGTTT

TGCCCCGATTGACTATCATGAAGTCGATTCCGCATTTGGTGACTGGGATGAC

GTAAAATGCTTGGGCGAAAAATACTACTTGATGTTTGATTTTATGATCAACC

ATATCTCACGTCAAAGCAAATACTACAAAGATTACCAGGAAAAACATGAAG

CTTCTGCCTACAAAGATCTGTTTCTTAACTGGGACAAATTTTGGCCGAAAA

ACCGGCCTACACAAGAAGACGTCGATCTTATCTACAAAAGAAAAGATCGC

GCCCCGAAACAAGAAATCCAGTTTGCAGACGGAAGCGTAGAACATTTATG

GAATACATTTGGTGAAGAACAGATTGACTTGGATGTTACAAAAGAAGTGAC

GATGGATTTTATCCGTTCTACGATCGAAAACTTGGCAGCGAACGGCTGTGA

TTTAATCCGGTTGGACGCATTTGCGTATGCTGTTAAAAAATTGGACACAAAC

GATTTCTTTGTGGAACCGGAAATTTGGACGTTATTGGACAAAGTTCGTGATA

TCGCTGCCGTGAGCGGAGCCGAAATCTTGCCTGAAATCCATGAACATTACA

CAATCCAATTTAAAATCGCGGACCATGATTATTACGTCTACGATTTTGCTCTG

CCGATGGTAACGCTGTATTCACTTTACTCAAGCAAAGTTGATAGACTGGCG

AAATGGCTTAAAATGAGCCCTATGAAACAGTTTACAACGTTAGACACACAT

GATGGTATTGGCGTTGTGGACGTGAAAGATATTCTGACGGATGAAGAAATC

ACATACACGTCAAACGAACTTTACAAAGTCGGCGCGAATGTAAACAGAAA

ATATAGCACAGCTGAATACAACAACCTGGATATCTACCAAATCAACTCTACG

TACTACTCCGCGCTTGGCGATGACGATCAGAAATATTTTCTGGCTCGCTTGA

TTCAAGCCTTTGCACCGGGAATCCCTCAGGTCTATTACGTAGGCTTTCTGGC

TGGAAAAAATGATCTTGAACTGCTTGAATCAACAAAAGAAGGACGTAACA

TCAACCGGCATTATTACTCTTCCGAAGAAATCGCCAAAGAAGTCAAAAGAC

CGGTCGTAAAAGCATTGTTGAACTTGTTTACGTACAGAAACCAAAGCGCAG

CGTTTGACTTGGATGGTCGCATTGAAGTTGAAACACCTAATGAAGCAACGA

TTGTGATCGAAAGACAGAACAAAGATGGATCTCATATTGCGAAAGCTGAAA

TCAATCTGCAAGATATGACATATCGCGTTACGGAAAACGACCAGACAATTT

CCTTTGAATAA

sucP

ATGAAAAACAAAGTACAGTTAATCACATACGCTGACCGGCTTGGCGACGGC

ACAATCAAATCCATGACAGACATCTTGCGGACACGCTTTGATGGCGTATATG

ACGGAGTTCATATTCTGCCGTTTTTCACGCCTTTTGATGGTGCGGACGCTGG

CTTTGATCCGATCGACCATACAAAAGTAGATGAACGTTTGGGCTCTTGGGA

TGACGTTGCCGAACTGTCCAAAACACATAATATTATGGTGGATGCAATCGTC

AACCATATGTCATGGGAAAGCAAACAATTTCAGGATGTACTTGCTAAAGGC

GAAGAATCTGAATACTACCCGATGTTTTTGACGATGTCAAGCGTCTTTCCTA

ATGGAGCGACAGAAGAAGATCTTGCTGGTATTTATAGACCGCGCCCTGGCT

TACCGTTTACGCATTACAAATTTGCCGGCAAAACAAGACTTGTTTGGGTTA

GCTTTACGCCTCAACAGGTCGACATCGATACAGACTCAGATAAAGGATGGG

AATACTTGATGAGCATCTTTGACCAGATGGCAGCGTCTCATGTATCCTATATC

CGCCTGGATGCAGTTGGCTACGGAGCCAAAGAAGCAGGCACAAGCTGCTT

TATGACACCGAAAACGTTTAAACTTATCTCTCGTTTACGGGAAGAAGGAGT

TAAACGTGGTTTGGAAATTCTGATCGAAGTGCATTCTTACTACAAAAAACA

AGTCGAAATCGCGTCAAAAGTTGATCGGGTGTATGACTTTGCGTTGCCGCC

TCTGCTTTTACATGCTCTGTCAACGGGACATGTCGAACCGGTAGCGCATTG

GACAGATATTAGACCTAATAACGCTGTCACAGTACTTGATACGCATGACGGT

ATCGGCGTGATTGATATCGGCTCTGATCAGTTAGACCGCTCCCTTAAAGGAT

TAGTCCCGGACGAAGATGTAGACAACTTGGTTAACACAATCCATGCTAACA

CGCATGGAGAATCTCAAGCTGCCACGGGTGCAGCGGCTTCCAATCTTGATT

TGTACCAGGTTAACTCAACATACTACAGCGCGCTGGGTTGTAATGATCAACA

TTACATCGCCGCACGTGCTGTGCAATTTTTCCTGCCGGGCGTTCCTCAGGTG

TATTACGTCGGCGCCCTGGCAGGAAAAAACGATATGGAATTGCTGCGGAAA

ACGAATAACGGCAGAGACATTAATCGCCATTATTACAGCACAGCCGAAATC

GATGAAAACCTTAAACGTCCGGTTGTGAAAGCATTGAATGCGCTGGCTAAA

TTTCGGAACGAATTAGATGCGTTTGACGGAACATTTTCATATACAACGGATG

ACGATACATCTATTTCCTTTACGTGGAGAGGTGAAACAAGCCAAGCGACAC

TTACGTTTGAACCGAAACGCGGACTTGGTGTGGATAATACAACGCCTGTCG

CCATGTTGGAATGGGAAGATTCAGCAGGAGACCATCGCAGCGACGATCTGA

TTGCCAACCCGCCTGTCGTAGCATAA
